# Relationship between Inflammatory and Biological Markers and Lung Cancer

**DOI:** 10.3390/jcm7070160

**Published:** 2018-06-25

**Authors:** Füsun Şahin, Ayşe Feyza Aslan

**Affiliations:** Department of Chest Diseases, University of Health Sciences/Yedikule Chest Disease and Thoracic Surgery Health Practice and Research Center, Istanbul 34760, Turkey; ayse_feyza@hotmail.com

**Keywords:** lung cancer, neutrophil/lymphocyte ratio, platelet/lymphocyte ratio, biological markers, cancer stage

## Abstract

We seek to define inflammatory markers, lipid and protein profiles that may aid in distinguishing lung cancer cases from those who are healthy and to determine the relationships between these levels and cancer stage and cell type. Lung cancer patients (*n* = 140, Group 1) and healthy cases (*n* = 50, Group 2) were enrolled. We retrieved platelet, platelet-associated markers (plateletcrit (PCT), mean platelet volume (MPV), platelet distribution width (PDW)), neutrophil/lymphocyte ratio-NLR, platelet/lymphocyte ratio-PLR, lipids (total cholesterol (TC), high density lipoprotein (HDL), low density lipoprotein (LDL), very low density lipoprotein (VLDL), triglycerides), proteins (total protein (TP) and albumin), and C-reactive protein (CRP) from electronic records and compared the data from lung cancer patients with those from healthy controls. Platelet, PCT, neutrophil, NLR, PLR, triglycerides, VLDL, and CRP levels were significantly higher in Group 1 compared with Group 2. MPV, lymphocyte, albumin, and HDL levels were significantly lower in Group 1 compared with Group 2. No significant relationship was evident between histopathological types and the level of any marker. Compared to those with early-stage cancer, changes in marker levels in those with advanced-stage cancer were statistically significant. CRP and NLR were significantly higher; albumin and HDL were lower in metastatic patients. We found that platelet, PCT, NLR and PLR, albumin, HDL, and CRP levels aided in lung cancer diagnosis and the detection of late-stage disease. Furthermore, these inflammatory and biological markers are thought to be particularly useful in following the severity of lung cancer.

## 1. Introduction

Lung cancer remains a major cause of mortality worldwide, principally because of its late diagnosis [1]. Inflammation plays a critical role in the progression of many cancers, stimulating cancer cell proliferation and angiogenesis [2]. Thus, various blood markers of inflammation have been evaluated in patients with different malignant tumors. These markers include platelet, neutrophil, and lymphocyte numbers but especially include neutrophil/lymphocyte ratio (NLR), platelet/lymphocyte ratio (PLR), mean platelet volume (MPV), plateletcrit (PCT), and platelet distribution width (PDW) [3,4,5]. Although changes in inflammation markers and lipid and protein profiles have been reported in lung cancer patients, the utility of such data remain unclear [6]. These inflammatory blood markers, lipid and protein levels can be routinely determined from complete blood counts and biochemical measurements which are inexpensive and reliable. In our study, we sought to define the inflammatory markers and lipid and protein profiles that may aid in the diagnosis of lung cancer, and we assessed the relationships of such measures with cancer stage and cell type.

## 2. Materials and Methods

One hundred and forty consecutive patients with lung cancer referred to Yedikule Chest Diseases and Thoracic Surgery Health Practice and Research Center between 2016 and 2017 (Group 1, male = 130, female = 10); age and gender matched 50 healthy controls (Group 2, male = 45, female = 5) were included in the study. 

The following patients were excluded: those with active infection or inflammation, hypertension, hematological or renal disease, heart failure, hepatic impairment; active bleeding; having a history of blood transfusion in the prior 3 months, acute myocardial infarction or cerebrovascular disease, a history of pulmonary embolism in the last month, a history of myeloproliferative disease, and autoimmune disease; as well as those who take steroids and have any other form of cancer. This analytical, case-control study was approved by the local ethics committee and written informed consent was obtained from all of the study participants. All study procedures were carried out in accordance with the Declaration of Helsinki.

Platelet numbers and the levels of platelet-associated markers (plateletcrit (PCT), mean platelet volume (MPV), platelet distribution width (PDW)), neutrophil/lymphocyte ratio-NLR, platelet/lymphocyte ratio-PLR, lipids (total cholesterol (TC), high density lipoprotein (HDL), low density lipoprotein (LDL), very low density lipoprotein (VLDL), triglycerides), proteins (total protein (TP) and albumin), and C-reactive protein (CRP) were measured. Preoperative or pretreatment blood data were obtained from electronic records. The NLR and PLR were calculated using these data. 

The results from lung cancer patients and healthy controls were compared. Cancer patients were subgrouped by stage (stage 1 = 11, stage 2 = 28, stage 3 = 52, stage 4 = 49), histopathological type (squamous = 55, adeno = 27, large cell = 1, undetermined type nonsmall cell lung cancer (NSCLC) = 32, small cell lung cancer (SCLC) = 25), and metastasis status (*n* = 34). The blood values of these subgroups were compared among themselves.

### 2.1. Measurements

Full blood counts were carried out using ABX Pentra 120 (Minnesota, USA); serum TC, HDL, LDL, VLDL, TG, TP, albumin, and CRP were analyzed with Olympus AU2700 Plus, Beckman Coulter (Tokyo, Japan) devices. 

### 2.2. Lung Cancer Staging

The TNM staging system was used for lung cancer staging. The TNM Staging System is based on the extent of the tumor (T), the extent of spread to the lymph nodes (N), and the presence of metastasis (M). The most up-to-date 8. TNM staging system was used in this study [7]. According to this staging system, stage IA, IB, IIA, IIB, and IIIA were considered early stage. IIIB, IIIC, IVA, and IVB were considered late (advanced) stage. Stage IVA and IVB were in the metastatic group.

### 2.3. Histopathological Types of Lung Cancer

Patients with lung cancer were classified to have squamous cancer, adeno cancer, undetermined type non-small cell lung cancer, small cell lung cancer, or large cell cancer. 

### 2.4. Statistical Analysis

All statistical analyses were performed using SPSS 16.0 statistical software package (SPSS Inc., Chicago, IL, USA). All numeric data were expressed as the mean ± standard deviation. Student’s *t*-test was used to evaluate the difference in platelet, platelet indices markers, NLR, PLR, protein and lipid levels between patients with lung cancer and healthy controls. The Kruskal Wallis Test was used in the comparison of histopathological types. The comparison of different stages and metastatic and non-metastatic cancer groups was analyzed using the Mann-Whitney U and Wilcoxon Tests. The correlation of CRP levels with lipid and protein concentrations was analyzed by the Mann-Whitney U Test. Inter-group comparisons were performed with a one-way analysis of variance. Correlations between numerical parameters were done with Pearson’s correlation test. Correlation between one numerical and one categorical or two categorical parameters was done with Spearman’s correlation test. Receiver operating characteristic (ROC) curve analysis was used to calculate the sensitivity and specificity of NLR and PLR in detecting lung cancer. A *p* value of <0.05 was accepted to be statistically significant.

## 3. Results

**Figure 1:**
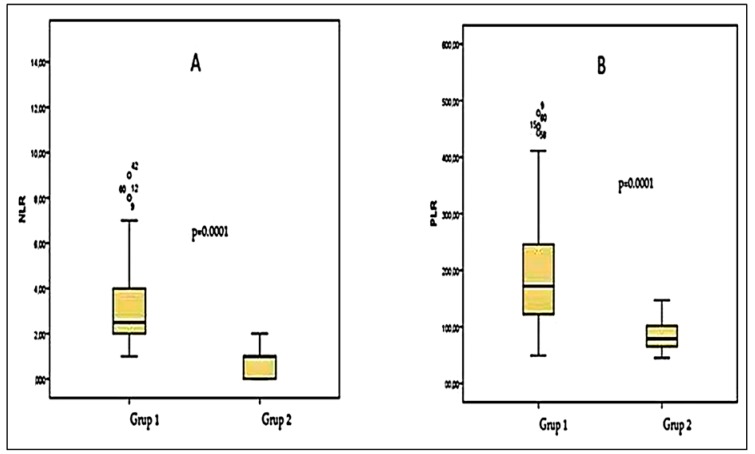
(**A**) The comparison of NLR (neutrophile/lymphocyte ratio) values in Group 1 (lung cancer patients) and Group 2 (control cases); (**B**) The comparison of PLR (platelet/lymphocyte ratio) values in Group 1 and Group 2.

**Figure 2:**
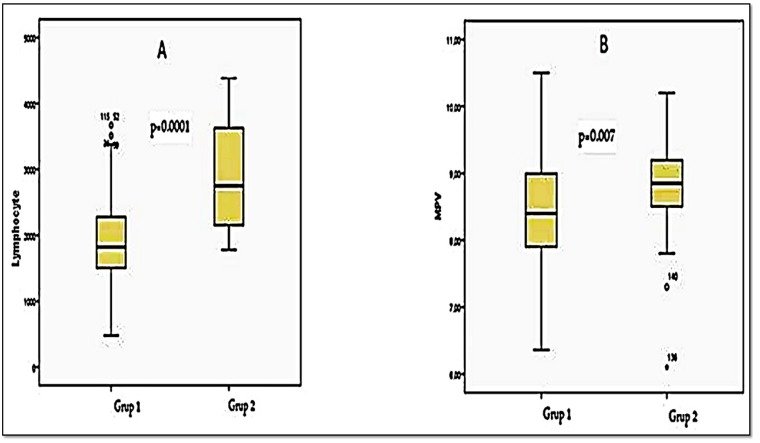
(**A**) The comparison of lymphocyte values in Group 1 (lung cancer patients) and Group 2 (control cases); (**B**) The comparison of MPV (mean platelet volume) values in Group 1 and Group 2.

Platelet numbers, PCT, neutrophil numbers, NLR (Figure 1A), and PLR (Figure 1B) were significantly higher in lung cancer patients than in healthy controls (*p* < 0.05, Table 1). The lymphocyte (Figure 2A) and MPV (Figure 2B) levels were significantly lower in patients with lung cancer than in controls (*p* < 0.05, Table 1). The PDW did not differ significantly between the groups (*p* > 0.05, Table 1). No significant relationship was evident between histopathological subgroup and any marker (*p* > 0.05, Table 2 and Table 3). According to the TNM stage, the associations of all markers (apart from the PDW) with advanced-stage cancer (stage 3 + stage 4, *n* = 101) were significantly greater than the associations with early-stage cancer (stage 1 + stage 2, *n* = 49) (*p* < 0.05, Table 2). In terms of metastasis status, the NLR was significantly higher in metastatic patients (*p* < 0.05, Table 3).

The albumin and HDL levels were significantly lower and the triglyceride, VLDL and CRP levels were significantly higher in cancer patients compared with control patients (*p* < 0.05, Table 1). The TP, TC, and LDL levels did not differ significantly between the groups (*p >* 0.05, Table 1). In those with advanced-stage (compared with early-stage) cancer, the albumin and HDL levels were significantly lower and the CRP level higher (*p <* 0.05, Table 2 and Table 3). These levels were not affected by the histopathological cancer type (*p >* 0.05, Table 2 and Table 3). The NLR and CRP levels were significantly higher. The albumin and HDL levels were lower in metastatic patients (*p <* 0.05, Table 3). 

**Figure 3:**
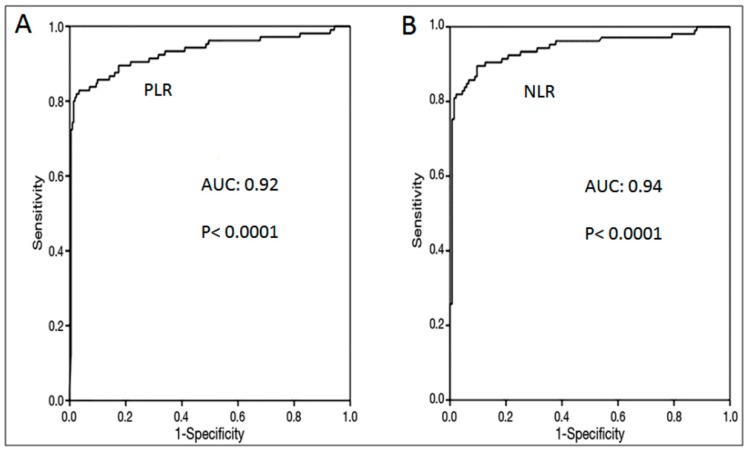
(**A**) ROC curve for using PLR (platelet/lymphocyte ratio) levels in the differential diagnosis of Group 1 (lung cancer patients) and Group 2 (control cases); (**B**) ROC curve for using NLR (neutrophil/lymphocyte ratio) levels in the differential diagnosis of Group 1 and Group 2.

ROC curve analysis was performed for the PLR and NLR values in detecting lung cancer patients. The best PLR cut-off value was defined as 112.5. For this cut off value, the sensitivity was 84% and the specificity was 90% (Table 4, Figure 3A). The best NLR cut-off value was defined as 1.5. For this cut off value, the sensitivity was 86% and the specificity was 92% (Table 4, Figure 3B).

## 4. Discussion

It is not known whether cancer affects erythrocyte, leucocyte, or platelet levels. The increase in leucocyte levels is partially attributable to the effects of cytokines [2]. Neutrophils, important components of inflammation, are suggested to play a significant inflammatory role in tumorigenesis [8]. Neutrophils support angiogenesis by secreting proangiogenic factors or proteolytic activation of such factors. Also, neutrophils ensure the collection of epidermal growth factor (EGF), transforming growth factor-β1 (TGF-β1), platelet-derived growth factors (PDGF), and other growth factors when contributing to tumorigenesis [9]. Neutrophils contain both pro- and anti-tumor subpopulations [10]. An increase in the neutrophil number was associated with a better prognosis in some studies and a poorer prognosis in others [11]. 

Chronic inflammation has been reported in patients with malignant tumors of epithelial origin (e.g., stomach, liver, colon, lung, pancreas, esophagus, bladder, and gallbladder cancers) [12]. Although the underlying mechanism remains unclear, active monocytes and neoplastic tissue have been suggested to trigger toxic granular inflammation mediated by neutrophils [13]. An increased neutrophil count was a strong independent prognostic factor for poor survival and relapse in patients with head-and-neck and kidney tumors [4,14]. However, an increased neutrophil count is not generally recognized to be associated with a poor cancer prognosis. Some studies found that high neutrophil counts were associated with good prognoses of gastric and low-grade prostate cancers [15]. In the present study, neutrophil counts were significantly higher in lung cancer patients than in healthy controls, and in late-stage lung cancer patients than in early-stage patients. 

Thrombocytes play significant roles in the growth, progression, and metastasis of cancer [16]. Hypercoagulability is a sign of aggressive disease, and thromboembolism is one of the principal causes of cancer mortality [17]. Thrombocytosis is common in lung cancer patients and is considered a reactive response to the carcinoma. This is also true of hemorrhage, hemolysis, infection, and inflammatory disease [18]. Although some studies found no relationship between the thrombocyte count and histopathological cancer type, associations were evident in other studies [19,20]. In our present study, the thrombocyte count was significantly higher in lung cancer patients (especially in late stage patients) than in controls but this was not associated with histopathological cancer type. The MPV has been evaluated in patients with different diseases of the vascular system (e.g., acute coronary syndrome, peripheral artery disease, deep vein thrombosis, and pulmonary thromboembolism) and many cancers. The MPV reflects platelet production in the bone marrow. If the MPV is high, platelet production is elevated; when it is low, platelet production is decreased. A high thrombocyte count combined with a low MPV indicates the presence of an infection, inflammation, or a malignancy [21,22]. Some studies found that the MPV, PCT, and PDW were higher or lower in patients with lung cancer than in healthy controls, but other works reported non-differing values [23,24,25]. In our study, the PCT was higher and the MPV lower in lung cancer cases than in controls, while the PDW did not differ significantly. Furthermore, cancer stage and histopathological type were not associated with the levels of any parameter assessed. 

In recent years, the NLR (reflecting high neutrophil levels during acute inflammation and secondary development of lymphopenia in response to acute physiological stress) has become recognized as a valuable index of the systemic immune response associated with inflammation and malignancy. An elevated NLR predicts a poor prognosis in cancer patients [3,26,27]. The PLR is another index of inflammation and the immune response associated with malignancy; both the NLR and PLR were elevated in lung cancer patients (as we also found) [5,28,29] but did not differ by cancer stage or histopathological type [29]. We found that the NLR and PLR were higher in those with late-stage cancer compared to those with early-stage cancer but this did not differ significantly based on histopathological type. 

Although hyperlipidemia is a negative prognostic factor in patients with stomach and prostate cancers, very few studies have explored the significance of this feature in lung cancer patients [30,31]. In one trial, the HDL, LDL, and TC levels were lower and the triglyceride levels higher in lung cancer patients compared with healthy controls; only the HDL level was prognostically significant [6]. A low HDL level was associated with elevations in proinflammatory cytokine production and chronic inflammation, while higher HDL levels mitigated both of these features. Thus, a reduction in the HDL level was associated with increased production of inflammatory markers including CRP [6]. We found that the HDL levels were low in cancer patients and the triglyceride and CRP levels high. 

## 5. Conclusions

The platelet, PCT, and especially the NLR, and PLR datas, assisted lung cancer diagnosis and the detection of advanced-stage disease and poor prognosis. Furthermore, the albumin, HDL, and CRP levels differed significantly in advanced stage lung cancer patients. These inflammatory and biological markers have been thought to be particularly useful in following the severity of lung cancer.

## Figures and Tables

**Table 1:** Laboratory findings of lung cancer patients (Group 1) and control cases (Group 2).

	Group 1 (mean ± SD)	Group 2 (mean ± SD)	*p* Value
Age (years)	58.85 ± 9.26	51.82 ± 8.99	0.14
Plt, ×10³/μL	330 ± 104.4	233 ± 371.8	0.03 *
MPV, fL	7.45 ± 0.80	8.79 ± 0.70	0.04 *
PCT, %	0.27 ± 0.07	0.21 ± 0.04	0.03 *
PDW, %	15.19 ± 2.24	14.77 ± 1.95	0.53
Neutrophile, μL	6548 ± 3141	3595 ± 768	0.01 *
Lymphocyte, μL	1928 ± 682	2924 ± 811	0.02 *
NLR	3.48 ± 0.31	0.76 ± 0.08	0.0001 *
PLR	105 ± 9.21	23.90 ± 3.38	0.0001 *
WBC, μL	9500 ± 3428	7100 ± 1313	0.72
Total protein, g/L	6.9 ± 0.7	7.0 ± 0.4	0.17
Albumin, g/L	3.5 ± 0.5	4.0 ± 0.3	<0.001 *
Total Cholesterol, mmol/L	163.7 ± 31	163.9 ± 21.9	0.93
Triglyceride, mmol/L	116.9 ± 42	100.9 ± 27.7	0.02 *
HDL, mmol/L	34.2 ± 7.7	47.7 ± 5.0	<0.001 *
LDL, mmol/L	104.3 ± 27	97.2 ± 20.2	0.15
VLDL, mmol/L	24.7 ± 12.3	19.9 ± 7.0	0.02 *
CRP, mg/L	12.9 ± 10.9	0.45 ± 0.2	<0.001 *

* Statistically significant, *p* < 0.05; SD, standard deviation; WBC, white blood cell; PLT, platelet; PDW, platelet distribution width; MPV, mean platelet volüme; PCT, plateletcrit; CRP, C-reactive protein; HDL, high-density lipoprotein; LDL, low-density lipoprotein; VLDL, very low-density lipoprotein; NLR, neutrophile/lymphocyte ratio; PLR, platelet/lymphocyte ratio.

**Table 2:** The relationship between laboratory findings and histopathological type and advanced- stage (TNM) in lung cancer group.

	Histopathological Type (*p* Value)	Advanced- Stage (TNM) (*p* Value)
Plt, ×10³/μL	0.23	0.02 *
MPV, fL	0.46	0.04 *
PCT, %	0.33	0.01 *
PDW, %	0.53	0.06
Neutrophile, μL	0.47	0.09
Lymphocyte, μL	0.49	0.02 *
NLR	0.59	0.03 *
PLR	0.34	0.001 *
Total protein, g/L	0.87	0.82
Albumin, g/L	0.16	0.02 *
Total Cholesterol, mmol/L	0.55	0.34
Triglyceride, mmol/L	0.32	0.76
HDL, mmol/L	0.80	0.001 *
LDL, mmol/L	0.60	0.98
VLDL, mmol/L	0.40	0.60
CRP, mg/L	0.78	0.004 *

* Statistically significant, *p* < 0.05; PLT, platelet; PDW, platelet distribution width; MPV, mean platelet volüme; PCT, plateletcrit; CRP, C-reactive protein; HDL, high-density lipoprotein; LDL, low-density lipoprotein; VLDL, very low-density lipoprotein; NLR, neutrophile/lymphocyte ratio; PLR, platelet/lymphocyte ratio.

**Table 3:** Correlations between laboratory parameters and other parameters in lung cancer group.

	NLR	PLR	HDL	Albumin	CRP
	*r*	*r*	*r*	*r*	*r*
Stage (TNM)	0.47 **	0.38 **	−0.32 **	−0.37 **	0.49 **
Histopathologic Type	NS	NS	NS	NS	NS
Metastasis	0.43 **	0.33 **	−0.35 **	−0.31 **	0.48 **
HDL	−0.29 *	−0.21 *		0.51 **	−0.39 **
Albumin	−0.32 **	−0.25 *	0.51 **		−0.35 **
CRP	0.42 **	0.36 **	−0.39 **	−0.35 **	

* *p* < 0.05; ** *p* < 0.001; r: correlation value. NLR, neutrophile/lymphocyte ratio; PLR, platelet/lymphocyte ratio; HDL, high-density lipoprotein; CRP, C-reactive protein.

**Table 4:** The sensitivity and specificity of NLR and PLR in detecting lung cancer.

	Ratio	Sensitivity (%)	Specificity (%)
NLR	1.5 *	86 *	92 *
	2	69	98
	2.5	50	100
PLR	106.5	85	82
	112.5 *	84 *	90 *
	120.5	78	92

* The best sensitivity and specificity values (cut-off point). NLR, neutrophile/lymphocyte ratio; PLR, platelet/lymphocyte ratio.
